# Knowledge and Attitude Toward Interpregnancy Interval and Its Impact on Women’s Health

**DOI:** 10.7759/cureus.104380

**Published:** 2026-02-27

**Authors:** Abeera Jamil, Hassan Mahmood

**Affiliations:** 1 Obstetrics and Gynecology, Lyell McEwin Hospital, Adelaide, AUS; 2 Master of Public Health, Western Sydney University, Sydney, AUS

**Keywords:** attitude, contraceptive use, family planning, interpregnancy interval, knowledge, maternal health, pregnancy spacing, women’s health

## Abstract

The interpregnancy interval, also known as pregnancy spacing, is one of the key factors influencing physical, mental, and reproductive health outcomes of women. According to the WHO, the minimum time interval between a live birth and succeeding conception is 24 months to minimize maternal and neonatal problems. Regardless of that, according to worldwide estimates, about 35-40% of pregnancies take place in intervals of less than 24 months, especially in low- and middle-income countries. This review employed a systematic search of PubMed and Google Scholar databases for peer-reviewed literature published between 2010 and 2025, using keywords related to interpregnancy intervals and maternal outcomes. Studies were included if they provided quantitative data on maternal morbidity and were excluded if they focused exclusively on neonatal outcomes or lacked clear diagnostic criteria. The lack of knowledge and negative attitudes toward pregnancy spacing remain key contributors to closely spaced pregnancies. This review explores what women know and how they feel about space during pregnancy and determines its effect on the health of women. Based on the available literature, short periods of interpregnancy (less than 18 months) are linked to the risk of maternal anemia, postpartum complications, and postpartum depression, with rates increased by 30-45%, 20-25%, and 15-20%, respectively. On the other hand, women who are well-informed and optimistic about pregnancy spacing record increased contraceptive use (ranging 50% and above) and improved maternal health markers (6-7 on clinical scales). The knowledge/attitude/pregnancy spacing interaction is critical to producing successful health promotion interventions. Optimal birth spacing can be strengthened by enhancing the quality of education, counseling, and engagement of healthcare providers, which can result in better health and well-being for women.

## Introduction and background

Interpregnancy intervals (IPIs) or pregnancy spacing is a major aspect of maternal and reproductive health. A good interval in between pregnancies gives women ample time to rest both physiologically and psychologically after pregnancy and birth. WHO has provided a 24-month gap between a live birth and the next conception interval (minimum) to lower the rates of poor maternal and baby health care [1]. Despite these guidelines, there is still a high occurrence of closely separated pregnancies in the world, and they are still in the way of presenting high challenges to the health of the population.

It is estimated that about 35-40% of pregnancies in the world are carried out in <24 months, and the highest rates are being witnessed in the low- and middle-income countries [2]. In South Asia and sub-Saharan Africa, the short birth intervals in almost one out of three women are a result of low access to family planning, poor reproductive health education, and sociocultural forces [3]. Closeness of pregnancies has been closely related to a high level of maternal morbidity due to reasons such as anemia, postpartum hemorrhage, and obstetric morbidity [4].

The information women have on the need to space their pregnancies is important in the determination of reproductive behavior. Research shows that women who have poor knowledge of optimal birth intervals are two to three times more likely to give close-distance pregnancies than women who have adequate knowledge [5]. Nevertheless, studies indicate that awareness of suggested spacing in pregnancy is not ideal, with the knowledge level reported to be between 30% and 60% in most developing areas [6]. The poor knowledge and low use of spacing methods are also brought about by misconceptions about contraceptive methods, fear of having side effects, and little counseling.

Women's attitudes toward pregnancy spacing have also been found to play a major part in making decisions besides knowledge. There is a cultural preference for large families, dominance of male partners, and the requirement of the society to have more than one child immediately after getting married, which in most cases leads to a negative view on birth spacing [7]. Research has established that 40-50% of women have negative attitudes toward pregnancy spacing despite their cognizance of its health advantages [8]. This has been cited as a significant impediment due to a lack of partner support, as about 30-40% of women stated that their partners were opposed to the use of contraceptives [9].

Health impacts of having shorter periods of pregnancy are not only based on physical impacts but also psychological and long-term reproductive impacts. The indication is that the women who had pregnancies that were close to each other are at increased risk of postpartum depression and chronic fatigue, which is 15-20% more than those who underwent the best spacing [10]. On the other hand, sufficient pregnancy spacing has also been linked to better maternal nutritional conditions, minimized obstetric problems, and enhanced overall quality of life [11].

The physiological strain of closely spaced pregnancies is primarily driven by "maternal depletion syndrome," where a short IPI prevents adequate replenishment of essential micronutrients, particularly iron and folate. This persistent nutritional deficit compromises maternal hematopoietic and immune recovery, significantly elevating the risk of gestational anemia and subsequent obstetric complications.

Knowledge, attitudes, and pregnancy spacing are some of the factors that need to be understood to come up with effective and efficient interventions to enhance the health of women. The focus of this review is to synthesize and provide available evidence on women's knowledge and attitudes toward spacing of pregnancies and how they affect physical, mental, and reproductive health outcomes. The emphasis on these aspects will help to develop specialized educational activities and healthcare policies to achieve the best pregnancy spacing and improve the well-being of women all over the world.

Background

The importance of pregnancy spacing in global maternal health has been of growing concern in the research on women because it is of much importance to women's health outcomes. In the past, reproductive health programs emphasized mainly fertility and child survival, but increasing statistics and evidence suggest the need to pay greater attention to proper birth intervals as a means of decreasing maternal morbidity and mortality [1]. Over the last 20 years, pregnancy spacing has been considered a cost-effective and sustainable approach to enhancing maternal health, especially in low- and middle-income nations.

Globally, an estimated 164 million women of reproductive age (15-49 years) have an unmet need for modern contraception. This gap is a primary driver of unintended pregnancies and closely spaced births, particularly in low-resource settings where satisfying this demand could reduce maternal deaths by nearly 30% [2]. Statistics indicate that almost 40% of all pregnancies around the globe are unintended, and a significant percentage of these are affected by short IPIs [3]. More than a third of women in some parts such as South Asia and sub-Saharan Africa give births after a period of less than 24 months, demonstrating the existing disparities in reproductive health provision [4].

Reproductive behavior has been found to largely depend on the knowledge and attitudes of women regarding pregnancy spacing. Surveys carried out in various locations indicate that knowledge of the suggested birth intervals is low, and only 30-60% of the women correctly reported the best spacing periods [5]. Moreover, attitudes against pregnancy spacing that are influenced by sociocultural constructs, gender relations, and the failure to tolerate the safety of contraceptives are still holding back the ability of the spacing practices [6].

The ill effects of improper spacing of pregnancies are well known. The shortness of the IPIs is related to the heightened risks of maternal anemia, obstetrics, and negative mental health consequences. It has been shown that women conceiving within a short interval are at the risk of up to 45% higher pattern of anemia and 20-25% greater chances of postpartum difficulty than their counterparts, who follow the advised interval between pregnancies [7,8]. Moreover, repeated pregnancies with insufficient time to rest may result in negative long-term health and quality of life for women due to the cumulative physical and psychological effects of pregnancy.

Even with the available family planning options, there exist discrepancies in education, access, and use. In most environments, male partners and extended family determine decisions on reproductive health, hence restricting freedom of spacing among women [9]. There is an indication that 30-40% of women cite the partner's resistance against using contraceptives, which means that comprehensive and culturally sensitive interventions are required [10].

A backdrop and contextual antecedents of pregnancy spacing are necessary in decoding women's knowledge and attitudes and their relationship with health outcomes. This background offers the basis to discuss the available evidence about the current practice of spacing during pregnancy and the means to enhance educational, counseling, and policy intervention that may help to improve the health of women.

**Figure 1:**
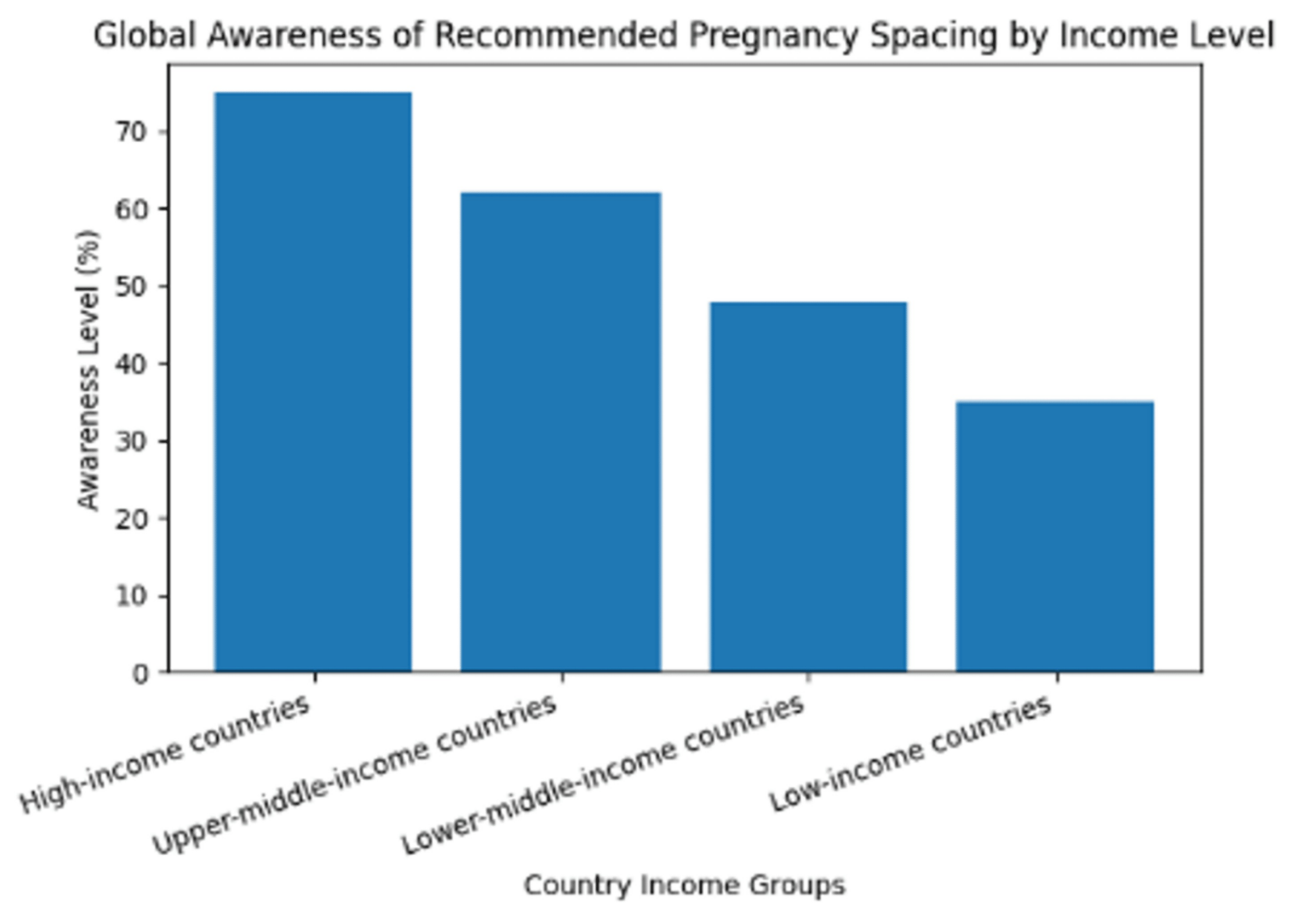
Global awareness of recommended pregnancy spacing by income level

Figure 1 shows changes in the level of awareness of the recommended pregnancy spacing among women across various country income groups in the world. The level of awareness is highest in high-income countries, then declines in upper-middle-, lower-middle-, and low-income countries. The figure reveals that there are some major differences in the knowledge of reproductive health that can cause the adoption of suboptimal pregnancy spacing among people, especially in resource-starved environments.

## Review

Materials and methods

The review synthesizes findings across various studies to identify consistent trends related to pregnancy spacing, maternal anemia, and neonatal outcomes. The inclusion and exclusion criteria are given in Table 1.

**Table 1:** Inclusion and exclusion criteria

Feature	Description
Databases	PubMed, Embase, Google Scholar, and WHO Global Health Library
Search terms	("Interpregnancy interval" OR "Birth spacing") AND ("Maternal anemia" OR "Postpartum depression") AND ("Knowledge" OR "Attitude")
Inclusion criteria	English language, peer-reviewed, published 2009-2024, and focuses on maternal morbidity/mortality
Exclusion criteria	Case reports, unpublished dissertations, and studies focusing solely on neonatal outcomes without maternal data

Concept of pregnancy spacing

Pregnancy spacing is the duration of time taken between the termination of one pregnancy and the next conception. It is the common exemption known as the birth interval and is known as one of the determinants of maternal and reproductive health outcomes. Enough time between pregnancies will enable women to rest between pregnancies and childbirth due to the physiological, nutritional, and psychological stress that women have during pregnancy. Pregnancy spacing is an important part of maternal and child health interventions that are highlighted by international health organizations [1].

While the WHO traditionally recommends a 24-month IPI to mitigate "maternal depletion," recent evidence suggests a more nuanced approach. Contemporary studies, such as those by the American College of Obstetricians and Gynecologists (ACOG), propose that 18 months may be the optimal balance, particularly for older maternal cohorts where the risks of delayed conception outweigh the benefits of prolonged spacing [1]. There is evidence indicating that the lower birth intervals below 18 months are characterized by very high rates of maternal complications. It was reported that women having short intervals between pregnancies were found to be at higher risk of maternal anemia (30-45%) [2,3] and at a greater risk of postpartum comorbidities, such as hemorrhage and infection, than those with optimal spacing [2,3].

Biologically short pregnancy intervals lead to maternal depletion syndrome, which manifests as a result of insufficient replacement of the body's nutrients such as iron, folate, and calcium. Such limitations may impair the immune functions of the mother and predispose her to negative health diseases [4]. Research has shown that women giving birth within 12 months after a previous birth have a high probability of demonstrating exhausted iron stores, and anemia may stem up to half of all populations in certain areas [5].

Besides the impact on physical health, pregnancy spacing has significant consequences for the psychological health of women. No time lapse between pregnancies leads to shortened bursts of recovery, greater burden of care, and higher degrees of stress, which can cause mental illnesses. It has been discovered that women within interpregnancy periods shorter than 18 months are at a risk of developing postpartum depression and chronic fatigue, 15-20% above ideal spacing [6].

On the other hand, the best pregnancy interval has been linked to favorable maternal health outcomes. Females with ≥24 months birth intervals have better nutrition status, reduced obstetric complications, and an improved quality of life [7]. Enough space also helps women use healthcare facilities, such as antenatal care and family planning, enabling them to make good reproductive decisions.

Even with properly laid-down rules, compliance with the recommended pregnancy spacing is not optimal. Socioeconomic inequality, accessibility to contraceptive tools, and insufficient knowledge on reproductive health services are reasons that tend to impede the best spacing practices, especially under conditions of resource deficiency [8]. The concept and importance of pregnancy spacing are thus peculiar to understand gaps in maternal healthcare and enhance effective long-term health outcomes for women.

Knowledge of pregnancy spacing among women

The information women have about spacing during pregnancy is a key indicator of reproductive health and the health outcomes for mothers. The knowledge here would mean being aware of the recommended intervals of births, health benefits of sufficient spacing, and knowledge of the available contraceptives. Several studies have shown that the lack of knowledge plays a significant role in close pregnancies and poor outcomes for mothers [1].

Global Awareness and Educational Disparities

The awareness with regard to recommended pregnancy spacing in the whole world is suboptimal. There are indications that 45-60% of women of reproductive age do not know that at least 24 months between the births of the baby is an ideal time to have more than one child [2]. The awareness level is even lower in low- and middle-income countries, especially in rural regions, where it is between 30 and 50% [3]. One of the significant factors that has been observed to lead to poor knowledge among these populations has been limited access to reproductive health education and counseling services [12].

Socioeconomic Determinants of Health Literacy

Learning status is an important factor in determining the information on pregnancy spacing among women. Research papers continue to show that educated women (who have been educated through secondary or higher education) tend to be much more informed on birth spacing than women who are not educated. Levels of awareness among educated women are more than 70%, and the level of knowledge among women with low education could be no more than 25-35% [4,13]. Also, women who regularly use antenatal and postnatal care services are better placed to be informed about the issue of spacing, and are 1.5-2 times more likely to practice the best pregnancy spacing [5,14].

Misconceptions and Barriers to Information

The misconceptions and misinformation are still prevalent despite the modern ways of birth control being available. About 30-40% of women are sure that long-term contraceptive use may cause infertility but about 25-30% are worried about the problem of having serious health conditions such as hormonal faltering or being ill. Such misconceptions have adverse impacts on knowledge and discourage the adoption of spacing methods even among women who are knowledgeable about the advantages of pregnancy spacing [6,15,16].

Many women rely on healthcare providers as a major source of information, but there are still less areas of counseling provision [17]. Research has also revealed that only half to three-quarters of women state that they were counseled adequately about the need to have rest between pregnancies in the course of the antenatal or postnatal check-ups [7]. The community programs of education and media campaigns have demonstrated potential in enhancing the level of knowledge, and it is documented that levels of awareness have increased by 20-30% after targeted intercessions [8,18].

Comprehensively, a lack of sufficient education about IPI is a major impediment to ideal reproductive health practices. It is imperative to strengthen education programs, offer better counseling services at regular check-ups, and counter misconceptions with the help of culturally sensitive measures to make women more knowledgeable and capable of spacing the pregnancies.

Table 2 summarizes regional variations in women’s knowledge of pregnancy spacing, including awareness of recommended birth intervals, understanding of contraceptive methods, and commonly reported knowledge gaps. Overall, knowledge levels remain lower in low- and middle-income regions than in high-income countries, highlighting the need for targeted educational interventions.

**Table 2:** Knowledge of pregnancy spacing among women across different regions Data are synthesized from peer-reviewed sources [1–30], with percentages representing ranges reported across the literature rather than a single dataset.

Region	Awareness of recommended birth interval (%)	Knowledge of contraceptive methods (%)	Major knowledge gaps identified
South Asia	45-55	50-60	Limited awareness of optimal interval and fear of side effects
Sub-Saharan Africa	30-50	40-55	Poor access to counseling and cultural misconceptions
Middle East and North Africa	60-70	65-75	Inconsistent counseling and partner influence
Latin America	65-75	70-80	Misinformation about long-term methods
High-income countries	75-85	80-90	Preference-based rather than health-based decisions

Attitude toward pregnancy spacing

The views of women concerning the spacing of pregnancies are critical determinants of reproductive behavior and use of family planning services. Attitude indicates what women believe, what they perceive, and their inclination to implement pregnancy-spacing practices, and it is usually what defines the experience of translating knowledge into action. Still, close conception and resulting health dangers can be caused by negative attitudes even among women who are aware of recommended periods of birth [1,19].

Sociocultural Norms 

It has been indicated that prejudice toward pregnancy spacing is still quite common in most areas. Research carried out in low- and middle-income states indicates that about 40-50% of women have negative perceptions about pregnancy spacing, basically because of the social culture and wrong beliefs about birth control methods [2,20]. Early, frequent childbearing that was preferred by the culture with relation to marriage, especially at a very young age, has a great effect on women and their understanding of time in pregnancy [3].

Male Partner Influence and Decision-Making Autonomy

The inclusion of male partners has been described as one of the key aspects in determining the attitude of women. Studies show that 30-40% of the female population exhibits partner opposition to taking steps to use pregnancy spacing methods as a significant obstacle [4,21]. Male partners or other extended family members of the female usually have the final say in the reproductive choices of the female in patriarchal societies, which curbs the independence of women and adversely impacts their feelings toward spacing [5,22]. On the other hand, it has been found that women whose partners support are almost twice as likely to develop positive attitudes and use optimum pregnancy spacing [6].

Contraceptive Apprehension

The other strong determinant of attitude is fear of contraceptive side effects. About 25-35% of women have doubts about the potential health risks of the contemporary contraceptive techniques such as hormonal imbalance, weight gain, and infertility [7,23]. These anxieties are often supported by falsehoods and poor counseling, which result in the reluctance to accept spacing practices, even after enlightenment on the benefits of the practice.

Attitudes that are favorable toward pregnancy spacing are closely linked to better health-seeking behavior. Females who hold positive perceptions have a higher use rate of contraceptives between 50 and >65 times than <30 times in females with a negative attitude [8,24]. Also, positive attitudes have been associated with greater use of antenatal and postnatal care services, which allow one to make informed choices related to reproductive decisions [9].

Attitudinal barriers should be addressed in order to lead to optimum pregnancy spacing. Male partner involvement, community-based interventions, and culturally sensitive counseling have proven to positively result in better attitudes by 20-30% in different settings [10]. By reinforcing these methods, it will be possible to adopt healthy spacing practices and improve maternal and reproductive health.

Table 3 highlights the determinants of the attitude of women toward pregnancy spacing and actual prevalence as reported. Significant contributors to negative attitudes are sociocultural norms, opposition of partners, and fear of contraceptive side effects, whereas education and supportive environments are pro-persuasive to the favorable attitude and acceptance of pregnancy spacing.

**Table 3:** Factors influencing women's attitude toward pregnancy spacing Data are synthesized from peer-reviewed sources [1–30], with percentages representing ranges reported across the literature rather than a single dataset.

Factor	Proportion of women affected (%)	Impact on attitude toward pregnancy spacing
Cultural and social norms	40-50	Promote early and repeated pregnancies
Male partner opposition	30-40	Reduced acceptance of spacing methods
Fear of contraceptive side effects	25-35	Negative perception of modern contraception
Limited counseling by healthcare providers	35-45	Inadequate attitude formation
Positive partner and family support	50-65	Improved acceptance of spacing practices
Exposure to reproductive health education	55-70	Favorable attitude toward spacing

Implication of pregnancy spacing on health of women

The interval between pregnancies is very powerful in influencing women's physical, mental, and long-term reproductive health. It has always been indicated that shorter IPIs have been linked to higher maternal morbidity, whereas optimal intervals help to enhance better health and well-being. There are more or less widespread implications of spacing between pregnancies on the health of women, which may be classified into physical, psychological, and reproductive outcomes.

**Figure 2:**
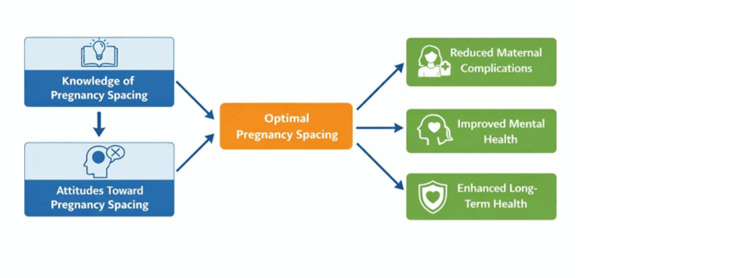
Conceptual framework linking knowledge and attitudes to pregnancy spacing and women’s health outcomes

Figure 2 demonstrates the conceptual relationship that accords women’s knowledge and attitudes toward pregnancy spacing and their roles in best practices for pregnancy spacing. This means that adequate knowledge and positive attitudes will result in optimal pregnancy spacing that subsequently results in better health outcomes in women, which include minimized maternal complications, better mental health, and better long-term reproductive and overall health.

Clinical health outcomes and maternal morbidity

The possibility of having the shortest pregnancy times, especially those under 18 months, is closely related to poor physical health outcomes. Research has shown that women who have close pregnancies are at a 30-45% higher risk of maternal anemia than women whose pregnancies are spaced well [1,25]. This risk is highly contributed to by the inefficiency in the process of replenishing iron and micronutrient stores after pregnancy and childbirth.

Women who have short birth intervals also have high postpartum complications. Studies indicate that conception trials occurring within 18 months of earlier births are associated with a 20-25% higher occurrence of postpartum bleeding and infection [2,26]. Moreover, evidence shows that short pregnancies increase the risk of hypertensive disorders by 15-20% [3]. These are some of the complications that lead to maternal morbidity, especially in the resource-restricted environment, where emergency obstetric care may be limited.

On the other hand, pregnancy spacing of 24 months or more has been taken to be advantageous in maternal nutritional status, obstetric complications, and a better recovery after giving birth. Women with proper spacing show reduced levels of anemia, better immune strength, and an increase in physical strength in subsequent pregnancies [4,27].

Impact on maternal psychological well-being

The psychological impacts of pregnancy spacing are becoming a more accepted issue in the study of maternal health. Closely spaced pregnancies put significant psychological pressure on the women through constraints in recovering and more caregiving. Literature indicates that suboptimal IPIs are a risk factor for maternal psychological distress, with studies showing a 15-20% increase in the incidence of postpartum depression among women with intervals shorter than 18 months [5,28].

Women who have close pregnancies also have higher chronic fatigue, emotional stress, and anxiety. Such mental issues can be compounded by socioeconomic stressors, absence of social support systems, and inaccessibility to mental health provisions [6,29]. On the contrary, sufficient pregnancy spacing has been linked to an enhanced emotional state, decreased stress levels, and enhanced coping potential in the postpartum period [7].

**Figure 3:**
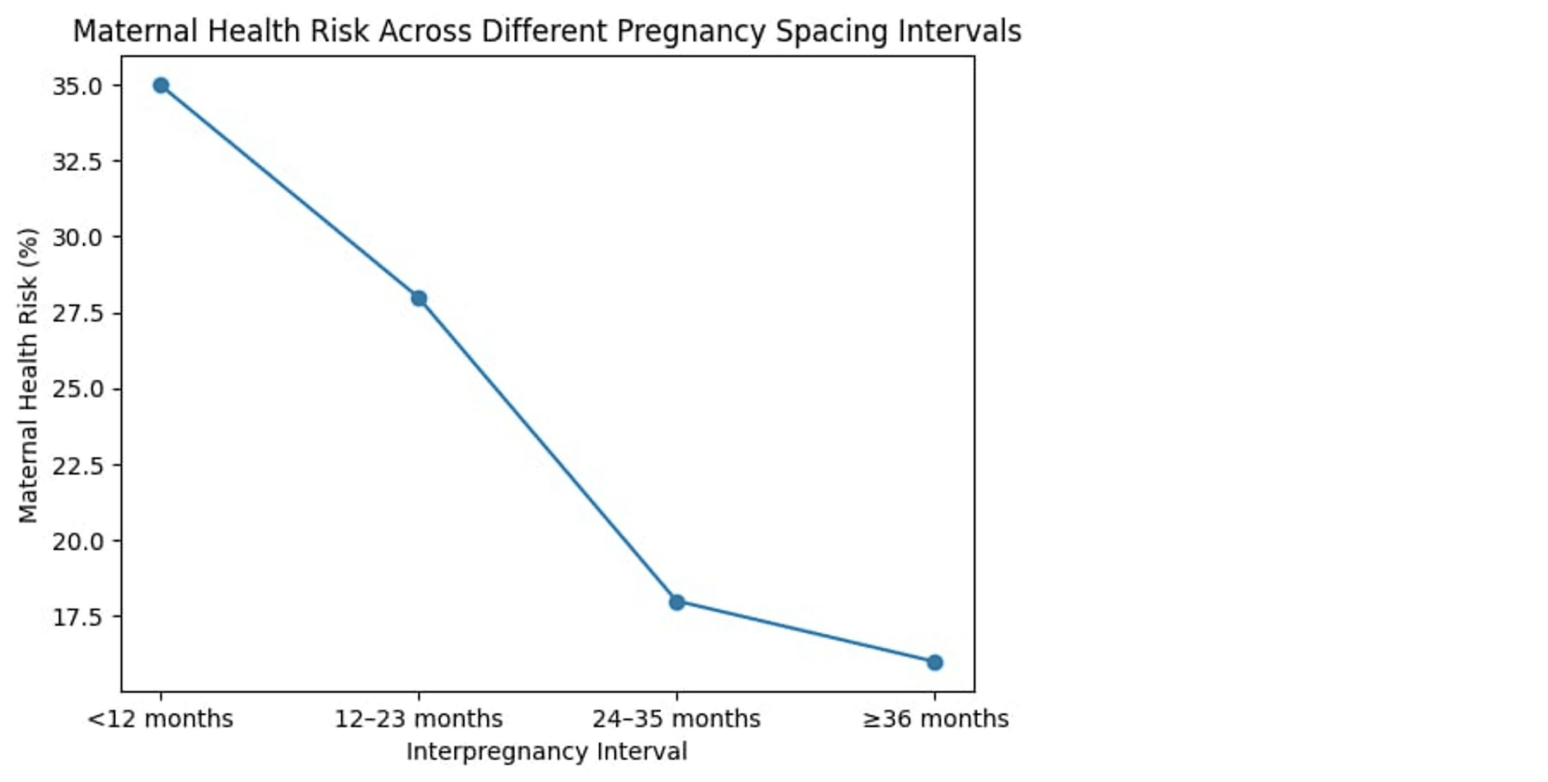
Maternal health risk across different pregnancy spacing intervals

Figure 3 shows the relationships between IPIs and maternal health risks. IPIs that are longer than a year are believed to increase the risk of negative maternal outcomes, whereas the optimal spacing of 24-35 months or more yields a significant reduction in maternal health risk. The figure underscores the safeguarding nature of a sufficient period between pregnancies on the health of women.

Long-term and reproductive health

Inadequate reproductive health of women can be compromised by short pregnancy spacing. Research has been able to establish that when pregnancies are closely spaced, it may lead to uterine complications such as poor uterine recovery and uterine rupture, especially in women whose deliveries had been made through cesarean section in the past [8,30]. Recurrent pregnancies without enough time for rest can also be a predisposing factor for chronic reproductive tract and pelvic floor dysfunction.

The best spacing between pregnancies, however, will help in the attainment of reproductive health and contribute to improved quality of life. Women following advice on spacing have better health, more inclusion in medical services, and more control over reproductive matters [9]. The long-term advantages of sufficient spacing are associated with a decrease in the cumulative health risks along with better maternal longevity.

Table 4 compares key health outcomes associated with short and optimal pregnancy spacing. Short IPIs are consistently linked to higher risks of physical and mental health complications, while optimal spacing is associated with improved maternal health and recovery.

**Table 4:** Impact of pregnancy spacing on women's health outcomes Data are synthesized from peer-reviewed sources [1–30], with percentages representing ranges reported across the literature rather than a single dataset.

Health outcome	Short spacing (<18 months)	Optimal spacing (≥24 months)
Maternal anemia	30-45% risk	Significantly reduced risk
Postpartum hemorrhage	20-25%	Lower incidence
Hypertensive disorders	15-20%	Reduced occurrence
Postpartum depression	15-20%	Improved mental well-being
Long-term reproductive health	Increased complications	Better recovery and outcomes

Table 5 highlights key strategies used to improve women’s knowledge and attitudes toward pregnancy spacing. Educational interventions, partner involvement, and healthcare system integration have demonstrated measurable improvements in spacing practices and maternal health outcomes.

**Table 5:** Strategies to improve knowledge, attitude, and practice of pregnancy spacing Data are synthesized from peer-reviewed sources [1–30], with percentages representing ranges reported across the literature rather than a single dataset.

Intervention strategy	Target population	Reported improvement (%)	Health impact
Antenatal and postnatal counseling	Pregnant and postpartum women	30-40	Increased awareness and contraceptive uptake
Community-based health education	Women of reproductive age	20-30	Improved knowledge and positive attitudes
Male partner involvement programs	Couples	25-35	Reduced partner opposition to spacing
Media and mass communication campaigns	General population	15-25	Reduced misconceptions about contraception
Integration of family planning into primary healthcare	Women accessing routine care	35-45	Improved spacing practices and maternal outcomes

Role of healthcare providers and education

The healthcare providers are at the heart of enhancing the level of knowledge and attitudes that women have toward pregnancy spacing and ensuring that they acquire the best birth spacing practices. Reliable health information providers have an advantage in providing accurate, evidence-based counseling during antenatal, postnatal, and routine reproductive health visits. Communication between healthcare professionals has been found to play a very important role in deciding how women will act regarding reproductive issues and the use of contraceptives [1].

Clinical Counseling

The positive news is that pregnancy spacing (contraceptive spacing) counseling given at both antenatal and postnatal care visits can boost contraceptive acceptance by 30-40% [2]. Female participants who undergo organized counseling by trained medical practitioners are one and a half or twice as likely to engage in the best practices in pregnancy spacing than their counterparts who have not received these counseling sessions [3]. Nevertheless, it is documented in the literature that only half to two-thirds of women remember getting proper information about the space between pregnancies by health workers, with specific reference being made to missed opportunities in the healthcare setup [4].

The healthcare center's mechanism of education is especially effective in the case of global misunderstandings and fears connected with contraception. It was found that fear of side effects decreases approximately 20-25% in cases of women under customized counseling and subsequent support [5]. Also, incorporation of pregnancy spacing education in the normal maternal and child health services has been linked to better continuity of care and greater use of family planning services [6].

Community-Based Interventions and Outreach Programs

The role of community health workers is also crucial in transferring education out of the healthcare facility, particularly in the underserved communities and rural regions. Educational interventions based within communities using trained health workers have shown a difference in knowledge and attitude of women to pregnancy spacing by 20-30% [7]. Such programs work especially well when they include home visits, group discussions, and culturally correct messaging.

Another major element of good education strategies is the presence of male partners. Literature suggests that male-inclusive reproductive health initiatives significantly mitigate partner opposition; such programs have reported a 30% reduction in resistance to modern methods, leading to more collaborative autonomy in determining optimal pregnancy intervals [8]. These strategies help to create more enabling conditions for women and improve the viability of the spacing practices.

On the whole, the additional role of medical personnel and the increased number of educational programs are crucial to advancing the ideal spacing between pregnancies. The knowledge, attitudes, and health outcomes of women concerning the issue of pregnancy spacing can be greatly improved with the help of improving the quality of provider training, ensuring uniformity in counseling practices, and incorporating family planning education into primary healthcare provisions [9].

Public health implications

Pregnancy spacing is a serious social health intervention that has distant outcomes on the health of women, healthcare systems, and population outcomes. Adequate birth spacing is a cost-effective measure that has been identified to minimize maternal morbidity and mortality, especially in low- and middle-income countries where healthcare resources are in most cases scarce [1].

Maternal mortality is one of the most significant issues of public health in the world, with approximately 287,000 registered maternal deaths due to childbirth every year. There is an indication that optimal pregnancy spacing may be able to prevent up to 10% maternal deaths by lowering the rate of pregnancy-related complications, including anemia, bleeding in childbirth, and high blood pressure disorders [2]. Little time between pregnancy periods causes further pressure on the available healthcare systems, since it leads to more emergency obstetric services and extended hospital care [3].

As to healthcare costs, a better depiction of the practices in pregnancy spacing can help to cut the cost of healthcare treatments that represent avoidable maternal complications. Research findings show that women who have the best pregnancy spacing use the antenatal care service better, undergo less emergency interventions, and make better use of healthcare resources [4]. Also, spacing leads to the enhancement of maternal nutritional status, which is long-term beneficial for the productivity and economic activities of women.

Pregnancy spacing is equally important for meeting global health targets, such as the Sustainable Development Goals associated with maternal health and gender equality. This act of educating women on the importance of chemical intervals during pregnancies and changing their attitudes toward these intervals will strengthen them in reproductive decision-making and enable them to have more autonomy in their reproductive choices [5]. Pregnancy spacing interventions incorporated into the primary health services of the public have been shown to increase contraceptive uptake by 25-35% and decrease the closely spaced pregnancies by up to 20% [6].

Interventions at the community and policy levels are critical in fueling the inequalities in pregnancy spacing. The increased availability of family planning services, enhanced training of healthcare providers, and male partner involvement can positively change people's health outcomes tremendously [7]. Mass media campaigns and education programs in schools also bring awareness and a positive attitude toward pregnancy spacing at the population level.

Overall, prioritizing pregnancy interval in public health agendas can lead to significant improvements in women’s health, particularly in reducing preventable maternal complications, and alleviating pressure on healthcare systems. The global health status is in need of comprehensive and culturally sensitive public health policies to promote pregnancy with optimal spacing and improve the quality of maternal health outcomes [8].

Future directions and recommendations

Although mounting evidence is pointing toward the advantages of optimal pregnancy spacing, there still appear to be a lot of discrepancies in women's knowledge, attitudes, and access to effective family planning services. Future directions must be in terms of empowering health systems, enhancing education, and combating sociocultural impediments toward ensuring sustainable practices in pregnancy spacing [1].

A major direction for the future is the incorporation of complete pregnancy spacing counseling into the mainstream process of maternal and child health. Research indicates that mandatory counseling procedures introduced in antenatal and postnatal services would make 30-40% more spacing behaviors optimal [2]. Education on the part of the healthcare provider to give consistent, culturally sensitive, and evidence-based information to the population is vital in incrementing quality of counseling and reducing the misconceptions linked to contraception.

Another significant strategy is expanding community-based interventions. Interventions such as community outreach, which are centered on trained health workers, have proved to increase the knowledge and attitude of women regarding pregnancy spacing by up to 30% [3]. Subserved and rural communities should be included in future programs because the level of awareness is minimal and access to healthcare is restricted. Digital health tools, including mobile health messaging and telehealth platforms, can also be used to improve the outreach and education process further.

The practice of male partner involvement in reproductive health programs should be a priority in the future. The evidence shows that when male partners are included in the family planning education, it is possible to lessen the opposition to the use of contraceptives by about 25-30% and also to enhance the joint decision of spacing of the pregnancies [4]. More helping environments with regard to the adoption of best spacing practices could be achieved by designing interventions that, instead of focusing on women alone, focus on couples.

Practices at the policy level play a vital role in maintaining progress as well. The national policies related to reproductive health put in place by governments and other health organizations targeting the general population should focus on the space between pregnancies, and they should invest enough in family planning initiatives. Providing access to cheaper contraceptive practices and maintaining supply chains will go a long way in curbing unmet need and unwanted pregnancies [5].

Lastly, additional studies would be required to identify context-related predictors of perceived knowledge and pregnancy spacing, especially among marginalized groups. Much evidence can assist in the policy and practice for those who study the benefits of optimal pregnancy spacing longitudinally, toward long-term health and socioeconomic advantages [6]. Each of these future directions can be addressed to enhance global initiatives on the healthy timing of pregnancy and improve the health outcomes of women.

## Conclusions

The interval between pregnancies is a very important factor that predetermines the physical, mental, and childbearing health of women. It is continuously proven that the risk of maternal anemia, postpartum complications, hypertensive disorders, and mental health difficulties is higher in cases of short IPIs, whereas the absence of risks is related to the following outcomes: adequate maternal well-being and recovery through optimal spacing of 24 months or higher. In spite of the clear recommendations that have been made globally, a significant percentage of women still encounter closely spaced pregnancies, given their lack of knowledge, negative attitudes, sociocultural factors, and restricted access to family planning services. This is evident in this review that the lack of proper awareness about recommended birth intervals and the use of misconceptions about the use of contraceptives are defining factors when it comes to women and their reproductive behaviors. Negative views influenced by cultural standards and the absence of partners also provide no assistance in the implementation of the best pregnancy spacing patterns. On the contrary, women who possess sufficient knowledge and positive attitudes have high contraceptive uptake and improved health outcomes.

Providers of healthcare and education are very much involved in providing solutions to these problems. Enhancing counseling in the course of antenatal and postnatal care, increasing community education initiatives, and enhancing the involvement of male partners are key measures in changing the practice of pregnancy spacing. Incorporating the concept of pregnancy spacing into mother health policies and helping to make the services of family planning fairly available to all can significantly decrease the maternal morbidity and mortality, which could be prevented in other ways. Finally, providing women with better knowledge and attitudes about spacing their pregnancies is an essential move toward coming up with better maternal health outcomes. All-embracing, culturally respectful, and evidence-based interventions must be promoted in order to facilitate the best pregnancy spacing and health and well-being of women in the heterogeneous population.
